# High-Performance Ag_2_Se Film by a Microwave-Assisted Synthesis Method for Flexible Thermoelectric Generators

**DOI:** 10.3390/molecules28176397

**Published:** 2023-09-01

**Authors:** Zixing Wang, Ying Liu, Jiajia Li, Changjun Huang, Kefeng Cai

**Affiliations:** Key Laboratory of Advanced Civil Engineering Materials of Ministry of Education, Shanghai Key Laboratory of Development and Application for Metal-Functional Materials, School of Materials Science & Engineering, Tongji University, Shanghai 201804, China; wzxing2023@163.com (Z.W.); liuying_polymer@163.com (Y.L.); sduljj2018@163.com (J.L.); hcj@tongji.edu.cn (C.H.)

**Keywords:** Ag_2_Se, thermoelectric, film, flexibility

## Abstract

Flexible Ag_2_Se thermoelectric (TE) films are promising for wearable applications near room temperature (RT). Herein, a Ag_2_Se film on a nylon membrane with high TE performance was fabricated by a facile method. First, Ag_2_Se powders were prepared by a microwave-assisted synthesis method using Ag nanowires as a template. Second, the Ag_2_Se powders were deposited onto nylon via vacuum filtration followed by hot pressing. Through modulating the Ag/Se molar ratio for synthesizing the Ag_2_Se powders, an optimized Ag_2_Se film demonstrates a high power factor of 1577.1 μW m^−1^ K^−2^ and good flexibility at RT. The flexibility of the Ag_2_Se film is mainly attributed to the flexible nylon membrane. In addition, a six-leg flexible TE generator (f-TEG) fabricated with the optimized Ag_2_Se film exhibits a maximum power density of 18.4 W m^−2^ at a temperature difference of 29 K near RT. This work provides a new solution to prepare high-TE-performance flexible Ag_2_Se films for f-TEGs.

## 1. Introduction

Flexible thermoelectric generators (f-TEGs) have attracted increasing attention due to their ability to harvest heat from irregular surfaces, including the human body, and turn it into electricity [1,2,3,4]. It is crucial to develop high-performance TE materials for fabricating excellent f-TEGs. The performance of a TE material is evaluated by the dimensionless figure-of-merit *ZT*. *ZT* = *S*^2^*σT*/*κ*, in which *S*, *σ*, *S*^2^*σ*, *T*, and *κ* stand for the Seebeck coefficient, the electrical conductivity, the power factor (PF), the absolute temperature, and the thermal conductivity, respectively [5,6].

Currently, conductive polymers and polymer-based composite films are mainly studied to fabricate f-TEGs [7,8,9]. Conductive polymers seem to be the first choice for developing F-TEGs, owing to their good flexibility and low *κ* value; however, a low PF value makes them difficult to use in high-performance devices. In comparison, inorganic TE films deposited on flexible substrates have more potential to realize a high TE performance and good flexibility. Recently, a (00l)-textured Bi_2_Te_3_ film on polyimide (PI) was reported to exhibit a high *ZT* of ~1.2 at room temperature (RT), as well as excellent flexibility [10]. Generally, f-TEGs are going to be used near RT. Although Bi_2_Te_3_ exhibits the best TE performance at RT, the toxicity and scarcity of Te is a matter of concern for application in f-TEGs.

Ag_2_Se is a promising Te-free n-type TE material thanks to its impressive TE performance near RT. Ag_2_Se is a narrow bandgap n-type semiconductor with a bandgap E_g_ = 0.07 eV at 0 K that has a phase transition from a low-temperature orthorhombic β-phase to a high-temperature cubic α-phase at around 407 K. Bulk β-Ag_2_Se has been reported to exhibit an ultrahigh PF ~ 3500 μW m^−1^ K^−2^ and *ZT* ~ 0.96 at RT, which is competitive with n-type Bi_2_Te_3_ [11]. In recent years, extensive strategies (such as stoichiometry manipulation [12,13], introducing a secondary phase [14,15], and doping [16,17]) have been adopted to optimize the TE properties of bulk Ag_2_Se and significant progress has been made. Nevertheless, bulk Ag_2_Se is rigid, even brittle, and hence may not be suitable for fabricating f-TEGs. Thus, many effective methods have been proposed to fabricate flexible Ag_2_Se films [18,19,20,21,22,23]. In 2019, our group first synthesized Ag_2_Se nanowires (NWs) via a wet chemical method, using Se NWs as a template and then combing them with vacuum-assisted filtration and hot pressing to achieve a flexible Ag_2_Se film on a nylon substrate; the film had a PF of 987 μW m^−1^ K^−2^ at RT [19]. Recently, Lei et al. [20] fabricated flexible Ag_2_Se films via the selenization of Ag films that were magnetron sputtered on PI in Na_2_S/Se mixed aqueous solution. The films exhibited an extremely high PF of ~2540 μW m^−1^ K^−2^, which is competitive with bulk Ag_2_Se. However, the prepared film was very thin (<2 μm), which is unfavorable for the output performance of assembled devices.

To further realize a higher PF for Ag_2_Se film, our group has adopted various approaches [24,25,26,27,28,29,30,31,32]. For example, Jiang et al. [24] raised the synthesis temperature of Ag_2_Se NWs from RT to 40 °C and the PF of the Ag_2_Se film was almost doubled (~1882 μW m^−1^ K^−2^). In addition, using the same preparation process and forming composite films with polyvinylpyrrolidone or polypyrrole, the films showed very high TE performances and ultrahigh flexibility [27,28]. However, the quality of Ag_2_Se NWs strongly relies on the Se NW templates and the synthesis steps for Se NWs take a long time, are easily affected by the ambient temperature, humidity, etc., and have poor repeatability. By contrast, the microwave-assisted synthesis method is effective and rapid for the preparation of nano-inorganic materials. Compared with conventional heating methods, the microwave-assisted synthesis method has many advantages, such as faster volumetric heating, a higher reaction rate, and a shorter reaction time [33,34]. For instance, Pei et al. [35] reported a rapid one-pot microwave-assisted solution method for fabricating Ag_2_Se dendrites; a maximum PF of 1300 μW m^−1^ K^−2^ was obtained at RT.

In this work, first, Ag_2_Se powder was prepared via a microwave-assisted synthesis method using Ag NWs as a template. Then, Ag_2_Se film was formed on a nylon membrane via vacuum-assisted filtration of the Ag_2_Se powder and hot pressing. A comparison of the Se NW template method and the Ag NW template method for the synthesis of Ag_2_Se powder is shown in Table S1 (Supplementary Materials). Compared with the Se NW template method, this method takes less time to synthesize Ag_2_Se powder (See Table S1). By adjusting the Ag/Se molar ratio, an optimized Ag_2_Se film is obtained that shows a high PF of 1577.1 μW m^−1^ K^−2^ at RT. A six-leg f-TEG was fabricated with the optimized Ag_2_Se film and its output performance was studied.

## 2. Results and Discussion

Figure S2a (Supplementary Materials) shows the X-ray diffraction (XRD) pattern of the Ag NWs. All the diffraction peaks are identified as the cubic Ag phase (JCPDS No. 04-0783). Figure S2b shows a typical scanning electron microscope (SEM) image of the Ag NWs. The Ag NWs have diameters ranging from 60 to 120 nm and lengths ranging from 5 to 10 µm.

The XRD patterns of powders 1–3 are depicted in Figure S3. The XRD pattern of powder 1 matches the orthorhombic Ag_2_Se (JCPDS no. 24-1041) phase. For powders 2 and 3, in addition to the orthorhombic Ag_2_Se phase, the cubic Ag phase (JCPDS no. 04-0783) is also present. As the Ag/Se molar ratio increases, the XRD peaks that are related to the Ag phase gradually become stronger.

The SEM images of powders 1–3 are presented in Figure S4. As shown in Figure S4a,b, powder 1 consists of particles with sizes of 0.1–1 μm. This suggests that the NW characteristics could not be maintained in the transition from Ag NWs to Ag_2_Se. This may be because cubic Ag and orthorhombic Ag_2_Se have different crystal structures and stress is generated in the Ag NW template when Ag is transformed into Ag_2_Se. When the stress accumulates to a certain degree, the Ag NWs break, which causes the Ag_2_Se to lose the NW characteristics. In addition, the microwave synthesis temperature is high and the reaction is violent, which are conditions that are not conducive to keeping the NW characteristics. Figure S4c,d and Figure S4e,f display the SEM images of powders 2 and 3, respectively. From Figure S4c–f, it is noted that powders 2 and 3 both show two different types of morphology, namely numerous particles with diameters of 0.2–1 μm and a few nanorods with a diameter of ~100 nm and a length of 1 μm, which correspond to the Ag_2_Se particles and Ag NWs, respectively.

**Figure 1:**
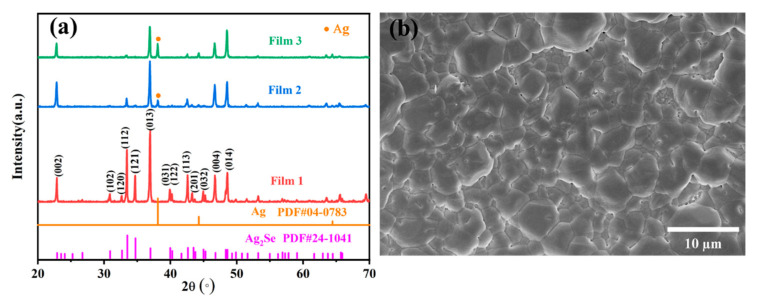
Characterization of the films. (**a**) XRD patterns of films 1–3. (**b**) Surface SEM image of film 1.

The XRD patterns of films 1–3 are depicted in Figure 1a. It can be observed that the XRD peaks of film 1 are in agreement with the standard orthorhombic Ag_2_Se phase. For films 2 and 3, the XRD peaks correspond to cubic Ag and orthorhombic Ag_2_Se, which reveals that both films are composed of Ag and Ag_2_Se. Increasing the Ag/Se molar ratio causes the Ag peaks to gradually become stronger, whereas the two strong peaks of the (112) and (121) planes of Ag_2_Se gradually weaken and even almost disappear in film 3. Figure 1b shows the surface SEM image of film 1. Film 1 possesses a dense microstructure with grain sizes of 1–9 μm and a few pores with sizes of 170 nm–375 nm in the grain boundaries. Figure S5 shows the cross-sectional SEM image of films 1–3, their average thicknesses are 11.4, 8.99, and 9.85 μm, respectively.

The X-ray photoelectron spectroscopy (XPS) analysis results for film 1 are given in Figure S6. As can be observed from the XPS survey spectrum (Figure S6a), Ag, Se, C, and O signals are detected; the C and O signals are caused by the sample being exposed to air. Figure S6b shows the Ag 3d spectrum, where the peak located at ~373.7 eV is assigned to Ag 3d_5/2_ and another peak at ~367.7 eV belongs to Ag 3d_3/2_, revealing the existence of Ag^+^. The Se 3d spectrum is presented in Figure S6c, in which the peaks of ~53.1 and ~53.9 eV correspond to Se 3d_5/2_ and Se 3d_3/2_, respectively, indicating the existence of Se^2−^. This confirms that the film consists of Ag_2_Se.

Figure S7a shows the TE properties of films 1–3 with different Ag/Se molar ratios at RT. As demonstrated in Figure S7a, with the Ag/Se molar ratio increasing from 2:1 to 3:1, the *σ* increases gradually from 769.4 to 1411.4 S cm^−1^ and the absolute *S* (|*S*|) decreases gradually from 143.2 to 101.7 μV K^−1^. As a result, film 1 exhibits a maximum PF of around 1577.1 μW m^−1^ K^−2^. The increase in *σ* and decrease in |*S*| are ascribed to the increasing Ag phase, with extremely high *σ* (10^5^ S cm^−1^) and relatively low |*S*| (5–7 μV K^−1^ [36]). The negative *S* indicates that they are all N-type conductions. Figure S7b displays the carrier concentration (*n*) and mobility (*μ*) at RT with various Ag/Se molar ratios. As the Ag/Se molar ratio rises from 2:1 to 3:1, the *n* increases from 4.52 × 10^18^ to 1.86 × 10^19^ cm^−3^; this is because the electrons of Ag are injected into the Ag_2_Se conduction band. Contrary to the trend for *n*, *μ* declines from 1061 to 474.1 cm^2^ V^−1^ s^−1^; this is caused by the low *μ* of Ag (10–25 cm^2^ V^−1^ s^−1^ at 300 K [37]). Generally, the *σ* is related to *n* and *μ,* namely, *σ* = *neμ.* Hence, the enhancement of *σ* is mainly because of the increase in *n*. For a semiconductor, the *S* has an inverse relationship with *n*, as written in Equation (1), where m_c_*, *k_B_*, *e*, and *h* denote the effective mass of the carrier, Boltzmann constant, electron quantity, and Planck constant, respectively.

(1) $$S=\left(\frac{8\pi^{2}k_{B}^{2}}{3eh^{2}}\right){m_{c}}^{*}T{\left(\frac{\pi }{3n}\right)}^{2/3}$$

Thus, it is readily understood that as the Ag/Se molar ratio increases, an increase in *n* leads to a decrease in |*S*|. Combined with the |*S*| and *n*, the density-of-states (DOS) effective mass (m*) of films 1–3 was calculated, as demonstrated in Figure S7c. For films 1–3, m* increases with the increasing Ag/Se molar ratio, which explains that the *μ* decreases; in the meantime, the *n* increases and the |*S*| decreases.

**Figure 2:**
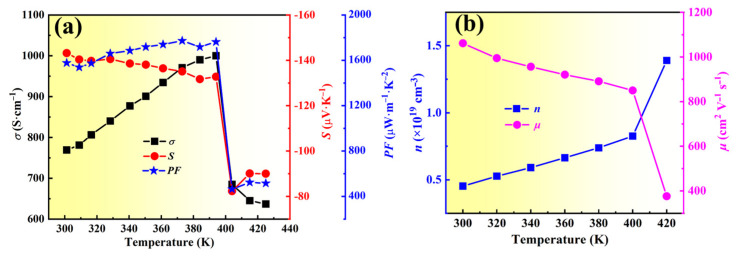
TE performance with the varying temperature of film 1. (**a**) *S*, *σ,* and PF. (**b**) *n* and *μ*.

Figure 2a presents the *S*, *σ,* and PF of film 1 measured from 300 to 420 K. As the temperature increases from 300 to 390 K, the |*S*| of the film declines from 143.2 to 132.7 μV K^−1^, whereas the *σ* of the film rises from 769.2 to 1000 S cm^−1^. As a result, the PF gradually increases from 1577.1 to 1762.1 μW m^−1^ K^−2^. Note that the *σ* and |*S*| decline rapidly as the temperature elevates from 390 to 410 K due to the phase transition from orthorhombic to cubic Ag_2_Se at 407 K. Figure 2b shows the *n* and *μ* of film 1 from 300 to 420 K. With increasing temperature under 400 K, the *n* exhibits an uptrend and the *μ* shows a downward trend. At 420 K, the *n* quickly increases to 1.39 × 10^19^ cm^−3^ and the *μ* sharply decreases to 376.0 cm^2^ V^−1^ s^−1^ due to the phase transition of Ag_2_Se. Thus, the variation in *σ* and *S* in response to temperature changes can be well understood by the Hall measurement results.

The thermal stability of film 1 was studied. The temperature dependence of the TE performance of film 1 for the heating and cooling cycle is shown in Figure S8. As *T* is in the range 300 to 400 K (below the phase transition temperature), the *S* and *σ* of the sample at a given temperature are basically the same during heating and cooling, indicating good thermal stability.

The temperature dependence of *S*, *σ*, and PF for films 2 and 3 is presented in Figure S9a and Figure S9c, respectively, showing that both films 2 and 3 exhibit an increase in *σ* and a decrease in |*S*| as the temperature increases. Figure S9b and Figure S9d show the temperature dependence of *n* and *μ* for films 2 and 3, respectively. Before 380 K, the *n* of both films 2 and 3 increases with the increasing temperature; the *μ* of film 2 is almost unchanged, whereas the *μ* of film 3 increases, which is different from the results for film 1. This may be related to the presence of the Ag phase in films 2 and 3.

**Figure 3:**
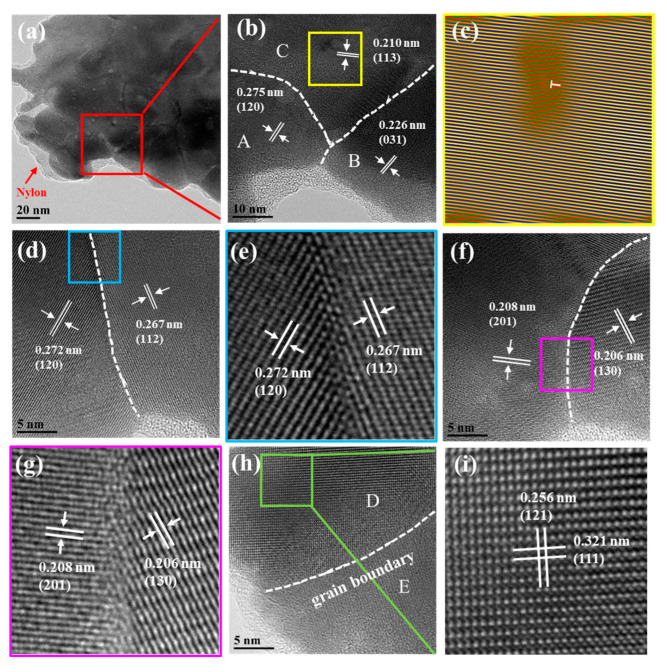
Internal microstructure of film 1. (**a**) A representative TEM image including a few nanograins. (**b**) HRTEM image corresponding to the square in (**a**). (**c**) IFFT image corresponding to the square in (**b**). (**d**) HRTEM image of two adjacent grains with a grain boundary. (**e**) Enlarged image of the square in (**d**). (**f**) HRTEM image of another two adjacent grains. (**g**) Enlarged image of the square in (**f**). (**h**) HRTEM image containing a well-crystallized grain and a poorly crystallized grain. (**i**) Magnified image of the square in (**h**).

To further investigate the detailed internal microstructure of film 1, transmission electron microscopy (TEM) analysis was performed; the results are shown in Figure 3. Figure 3a shows a representative TEM image of a few nanograins with sizes ranging from 40 to 100 nm, which also are observed in Figure S10a. The size of the nanograins is smaller than that of Ag_2_Se powder (see Figure S4a), meaning that the powder has undergone a melting, nucleation, and recrystallization process. Note that an amorphous layer is observed at the edge of the Ag_2_Se grains that was deduced to be nylon, which is also found in Figure S10a. This is because the Ag_2_Se film is tightly bonded with nylon and the nylon tightly bonded with the Ag_2_Se grains was also scraped off during the preparation of the TEM sample. Furthermore, some stacking faults in grains are observed in Figure S10a,b. Figure 3b is a magnified image of the red square marked in Figure 3a, in which the three Ag_2_Se grains, A, B, and C, form a triangle boundary and have different orientations, including the (120), (031), and (113) planes of orthorhombic Ag_2_Se, respectively. Likewise, Figure S10c shows a triangle grain boundary formed by three Ag_2_Se grains, G, H, and I. To better observe the triangular grain boundary, a magnified image is shown in Figure S10d. It can be seen that atoms at the triangular grain boundaries are irregularly arranged. The lattice spacing of grain G and grain H are 0.271 and 0.266 nm, respectively, corresponding to the (120) and (112) planes, whereas grain I shows poor crystallinity. Figure 3c shows an inverse fast Fourier transform (IFFT) image corresponding to the yellow square marked in Figure 3b. A distinct edge dislocation is observed in Figure 3c. Figure 3d displays a high-resolution TEM (HRTEM) image of two adjacent grains with (120) and (121) planes. An HRTEM image at higher magnification is shown in Figure 3e to display the detail of the grain boundary. The atoms at the grain boundary are regularly arranged, with no defects or impurities. Figure 3f shows an HRTEM image of two adjacent grains with (201) and (130) planes and a grain boundary. The enlarged HRTEM image (Figure 3g) more clearly shows that the atoms at the grain boundary are somewhat disordered. Figure 3h presents an HRTEM image of a well-crystallized grain, grain D, and a poorly crystallized grain with a grain boundary, grain E. To better show the crystallinity of grain D, a magnified image is presented in Figure 3i that shows that the atoms are regularly arranged, indicating excellent crystallinity. Grain D exhibits lattice spacing of ~0.256 and 0.321 nm, which are assigned to the (121) and (111) planes of Ag_2_Se, respectively. Likewise, Figure S10e shows an HRTEM image of another two adjacent grains, J and K. It can be seen that some stacking faults in grain J are near the grain boundary. An enlarged image of the blue square marked in Figure S10e is shown in Figure S10f, which shows that the atoms in grain K are irregularly arranged, revealing poor crystallinity. The lattice spacing of grain J is 0.250 nm, corresponding to the (013) plane of Ag_2_Se.

Here, the *κ* of the film was not measured, as the Ag_2_Se film is difficult to peel off from the nylon. The *κ* comes from two sources: electrical thermal conductivity (*κ_e_*) and lattice thermal conductivity (*κ_l_*). The Lorenz (L) number is calculated to be 1.74 × 10^−8^ W Ω K^−2^ by using the single parabolic band (SPB) model. The *κ*_e_ (=L*σ*T) is 0.403 W m^−1^ K^−1^ at 300 K, which is lower than that (*κ*_e_ = 0.470 W m^−1^ K^−1^) of the reported Ag_2_Se film on nylon in ref. [23]. It is believed that the *κ_l_* of film 1 is low for the following reasons: film 1 consists of grains with sizes ranging from 40 nm to 9 μm with many grain boundaries and some grains contain stacking faults and/or edge dislocations that can effectively scatter phonons and hence lower the *κ_l_*. Therefore, it is deduced that the *κ* of film 1 is smaller than that (*κ* = 0.78 W m^−1^ K^−1^) for the Ag_2_Se film with fewer defects reported in ref. [23], namely, the *ZT* of film 1 is estimated to be >0.6 at RT.

**Figure 4:**
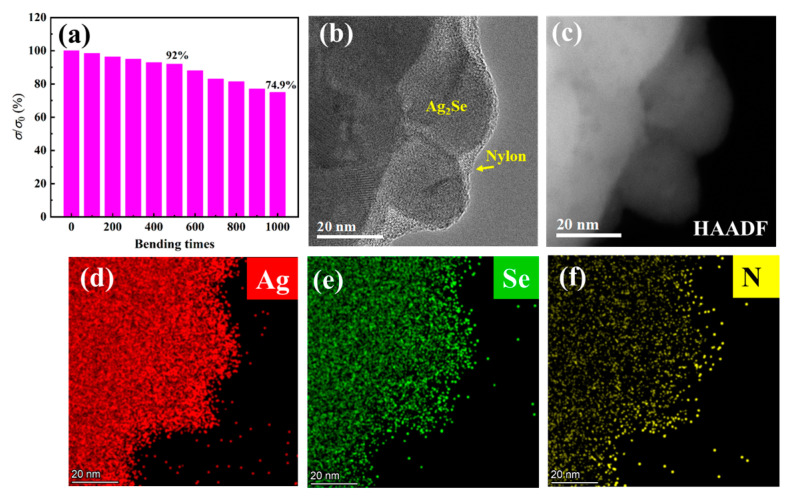
Flexibility of the film and evaluation of the Ag_2_Se/nylon heterointerface. (**a**) Flexibility test of film 1. (**b**) TEM image of Ag_2_Se grains with an amorphous nylon layer. (**c**) HAADF image corresponding to (**b**). (**d**–**f**) corresponding EDS mapping images of elemental Ag, Se, and N.

Flexibility is another important parameter for flexible TE film. Therefore, the flexibility of film 1 was tested. The film was bent with a 4 mm radius for a different number of times and the corresponding *σ* was measured, as depicted in Figure 4a. The *σ* of film 1 decreases with an increasing number of bending times; it retains 92% and 74.9% of the original *σ* after bending 500 and 1000 times, respectively. The flexibility of film 1 is mainly derived from the flexible nylon substrate. Figure 4b presents a TEM image of a few Ag_2_Se grains with an amorphous nylon layer, which is also observed in Figure 3a,b and Figure S10a. The Ag_2_Se grains and the nylon membrane are well bonded, which is beneficial for flexibility. Figure 4c–f shows a high-angle annular dark field (HAADF) image of Ag_2_Se grains with amorphous nylon membrane, corresponding Ag, Se, and N element energy dispersive spectroscopy (EDS) mapping images, respectively. As seen from the EDS mapping images of elemental Ag and Se (Figure 4d,e), the Ag and Se elements are homogeneously distributed. Figure 4f displays the EDS mapping image of elemental N and shows that the N element is enriched at the edge of the Ag_2_Se grains, which comes from the CONH group of the nylon membrane.

Generally, the smaller the grains (more grain boundaries) in a film, the more difficult it is for a crack to propagate. This is due to more energy consumption at the grain boundary, namely, the better flexibility of the film. Compared with the Ag_2_Se film reported in ref. [19], film 1 has larger Ag_2_Se grains (with some grain sizes reaching 9 μm), which results in its relatively lower flexibility.

**Figure 5:**
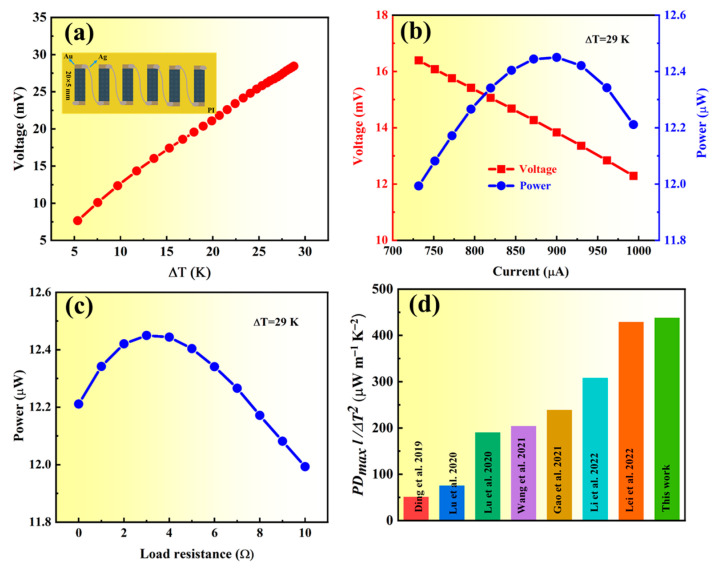
The output performance measuring result of the f-TEG. (**a**) Open-circuit voltage vs. ΔT. (**b**) *V_out_* and *P* vs. current at ΔT = 29 K. (**c**) *P* vs. load resistance at a ΔT = 29 K. (**d**) Comparison of the *PD_max_ l*/ΔT^2^ value of the f-TEG and that of some reported f-TEGs at a similar a ΔT [19,20,25,26,29,30,32].

To demonstrate the power generation capability of film 1, a six-leg f-TEG was fabricated. Figure S11 presents a circuit schematic for measuring the performance of the f-TEG. One end of the f-TEG is kept at RT, the other end is heated to a specified temperature; then, the output performance of the f-TEG is tested using a homemade apparatus. The open-circuit voltage (*V_oc_*), as a function of temperature difference (ΔT), is depicted in Figure 5a, showing that the f-TEG generates a *V_oc_* of 28.3 mV when ΔT = 29 K. The output power (*P*) is calculated by using Equation (2), where *R_load_* and *R_in_* represent the output voltage, load resistance, and internal resistance of the f-TEG.

(2) $$P={\left(\frac{V_{oc}}{R_{load}+R_{in}}\right)}^{2}\times R_{load}$$

The maximum output power (*P_max_*) is gained when *R_load_* matches *R_in_*. Figure 5b shows the output voltage (*V_out_*) and P versus the output current at ΔT = 29 K. The *V_out_*, output current, and *P_max_* are 13.83 mV, 900 μA, and 12.44 μW, respectively (see Figure 5b), and the corresponding *R_load_* is 3 Ω (Figure 5c). The maximum power density (*PD_max_*) is calculated using Equation (3), where *N* and *A* represent the number of legs and the cross-sectional area of the leg, respectively.

(3) $${PD}_{max}=\frac{P_{max}}{N\cdot A}$$

As a result, a *PD_max_* of 18.4 W m^−2^ is acquired by the present f-TEG at a ΔT of 29 K, which is significantly larger than the value (2.3 W m^−2^) for the f-TEG at a similar ΔT reported in ref. [19]. The comparison of the normalized *PD_max_* (*PD_max_ l*/ΔT^2^) [24] value of the f-TEG and that of some reported f-TEGs at a similar ΔT is shown in Figure 5d. As shown in Figure 5d, the normalized *PD_max_* of the f-TEG reaches 438.3 μW m^−1^ K^−2^, which is better than that of previous f-TEGs based on Ag_2_Se-based film at a similar ΔT.

## 3. Materials and Methods

Ethylene glycol (EG) (≥99.0%), ethanol (≥99.7%), iron trichloride hexahydrate (FeCl_3_·6H_2_O) (≥99.0%), copper(Ⅱ) chloride dehydrate (CuCl_2_·2H_2_O) (≥99.0%), polyvinylpyrrolidone (PVP) K-30, sodium dodecyl benzene sulfonate(SDBS) (≥88.0%), silver nitrate (AgNO_3_) (≥99.8%), selenium (Se) powder (≥99.0%), and hydrazine hydrate (N_2_H4·H_2_O) (≥85.0%) were purchased from Sinopharm Chemical Reagent Co., Ltd., China. All the reagents were used directly without further purification. A porous nylon membrane with diameter of 50 mm and pore diameter of 0.22 μm was purchased from Haiyan Taoyuan Group. The deionized (DI) water used for all experiments was from a Flom ultrapure water system. The steps involved in preparing Ag NWs are shown in Note S1 (Supplementary Material). The schematic of the preparation process of the Ag_2_Se films is depicted in Figure S1 (Supplementary Material). In detail, different contents of Se powder and 10 mL N_2_H_4_·H_2_O were added into a Ag NW and ethylene glycol (EG) dispersion (nominal molar ratio of Ag: Se = 2:1, 2.4:1 and 3:1, respectively) with slow stirring in a 200 mL beaker. Then, the obtained solution in a beaker was placed into a domestic microwave oven and reacted at 350 W for 5 min. After the reaction, the black product was collected by centrifugation and then washed repetitively with absolute ethanol and distilled water. The Ag_2_Se powder was dispersed in ethanol and then vacuum filtered on a nylon membrane to form a film. After drying, the film on nylon was then first cold pressed at 30 MPa, followed by hot pressing at 230 °C and 1 MPa for 30 min. For simplicity, the powders and films obtained from Ag/Se molar ratios of 2:1, 2.4:1, and 3:1 were named powders 1–3 and films 1–3, respectively.

A TEM sample was prepared as follows: a piece of film was scraped from the nylon substrate, ground in alcohol with an agate mortar for 1 h, sonicated in alcohol for 30 min, and then finally dropped on a microgrid.

The fabrication of the f-TEG is given in Note S2 (Supplementary Material). The related characterization and measurements are described in Note S3 (Supplementary Material).

## 4. Conclusions

In this work, a flexible Ag_2_Se film on nylon was prepared by combining microwave-assisted synthesis, vacuum filtration, and hot pressing. The TE property was optimized by adjusting the Ag:Se ratio for the synthesis of Ag_2_Se powder. The optimized film is dense and consists of grains with sizes ranging from 40 nm to 9 μm, as revealed by SEM and TEM observation, and the film exhibits a PF of ~1577.1 μW m^−1^ K^−2^ at RT. The flexibility of the Ag_2_Se film is mainly derived from the flexible nylon substrate. A six-leg f-TEG assembled with the optimal film generates a 28.3 mV voltage and 12.44 μW power (corresponding power density ~18.4 W m^−2^) under a temperature gradient of 29 K. This work demonstrates a new route to high-TE-performance flexible Ag_2_Se films on nylon.

## Data Availability

Not applicable.

## Supplementary Materials

The following supporting information can be downloaded at: https://www.mdpi.com/article/10.3390/molecules28176397/s1, Note S1: Synthesis of Ag NWs; Note S2: Assembly of the flexible TE generator; Note S3: TE property measurement and structure characterization; Note S4: Theoretical calculation details; Figure S1: Schematic demonstrating the preparation process of the Ag2Se film; Figure S2: Characterization of the Ag NWs. (a) XRD pattern of Ag NWs, (b) surface FESEM image of Ag NWs; Figure S3: XRD patterns of powders 1–3; Figure S4: Surface FESEM images of powders 1–3: (a,b) powder 1, (c,d) powder 2, (e,f) powder 3; Figure S5: Cross-sectional SEM images of films 1–3: (a) film 1, (b) film 2, (c) film 3; Figure S6: XPS analysis of film 1. (a) XPS survey spectrum. (b) Ag 3d spectrum. (c) Se 3d spectrum; Figure S7: TE performance at RT of films 1–3 as functions of the nominal molar ratios of Ag/Se. (a) S, σ, and PF. (b) n and μ. (c) Hall carrier concentration dependence of the Seebeck coefficient with estimated DOS effective masses for the three films; Figure S8: Temperature dependence of the TE performance of film 1 for the heating and cooling cycle. (a) σ, (b) S; Figure S9: TE performance with varying temperature for films 2 and 3. (a) S, σ, and PF of film 2. (b) n and μ of film 2. (c) S, σ, and PF of film 3. (d) n and μ of film 3; Figure S10: Internal microstructure of film 1. (a) Representative TEM image including a few nanograins. (b) HRTEM image corresponding to the pink square marked in (a). (c) HRTEM images corresponding to the green square marked in (a). (d) Enlarged image of the yellow square marked in (c). (e) HRTEM image of another two adjacent grains. (f) Enlarged image of the blue square marked in (e); Figure S11: A schematic diagram for the output performance measurement of the f-TEG; Table S1: Comparison of the two methods of synthesis for Ag2Se powder.

## References

[1] Li X., Cai K., Gao M., Du Y., Shen S. (2021). Recent advances in flexible thermoelectric films and devices. Nano Energy.

[2] Cao T., Shi X.-L., Chen Z.-G. (2023). Advances in the design and assembly of flexible thermoelectric device. Prog. Mater. Sci..

[3] Yang S., Qiu P., Chen L., Shi X. (2021). Recent Developments in Flexible Thermoelectric Devices. Small Sci..

[4] Zhang L., Shi X.-L., Yang Y.-L., Chen Z.-G. (2021). Flexible thermoelectric materials and devices: From materials to applications. Mater. Today.

[5] Zhao W., Liu Z., Sun Z., Zhang Q., Wei P., Mu X., Zhou H., Li C., Ma S., He D. (2017). Superparamagnetic enhancement of thermoelectric performance. Nature.

[6] Wang Y., Yang L., Shi X.-L., Shi X., Chen L., Dargusch M.S., Zou J., Chen Z.-G. (2019). Flexible Thermoelectric Materials and Generators: Challenges and Innovations. Adv. Mater..

[7] Liu S., Li H., Li P., Liu Y., He C. (2021). Recent Advances in Polyaniline-Based Thermoelectric Composites. CCS Chem..

[8] Song H., Meng Q., Lu Y., Cai K. (2019). Progress on PEDOT:PSS/Nanocrystal Thermoelectric Composites. Adv. Electron. Mater..

[9] Xu S., Shi X.-L., Dargusch M., Di C., Zou J., Chen Z.-G. (2021). Conducting polymer-based flexible thermoelectric materials and devices: From mechanisms to applications. Prog. Mater. Sci..

[10] Zheng Z.-H., Shi X.-L., Ao D.-W., Liu W.-D., Li M., Kou L.-Z., Chen Y.-X., Li F., Wei M., Liang G.-X. (2023). Harvesting waste heat with flexible Bi_2_Te_3_ thermoelectric thin film. Nat. Sustain..

[11] Ferhat M., Nagao J. (2000). Thermoelectric and transport properties of β-Ag_2_Se compounds. J. Appl. Phys..

[12] Lee C., Park Y.-H., Hashimoto H. (2007). Effect of nonstoichiometry on the thermoelectric properties of a Ag_2_Se alloy prepared by a mechanical alloying process. J. Appl. Phys..

[13] Wang P., Chen J.-L., Zhou Q., Liao Y.T., Peng Y., Liang J.S., Miao L. (2022). Enhancing the thermoelectric performance of Ag_2_Se by non-stoichiometric defects. Appl. Phys. Lett..

[14] Wang H., Han G., Zhang B., Chen Y., Liu X., Zhang K., Lu X., Wang G., Zhou X. (2023). AgSbSe_2_ inclusions enabling high thermoelectric and mechanical performance in n-type Ag_2_Se-based composites. Acta Mater..

[15] Chen J., Sun Q., Bao D., Tian B.-Z., Wang Z., Tang J., Zhou D., Yang L., Chen Z.-G. (2021). Simultaneously enhanced strength and plasticity of Ag_2_Se-based thermoelectric materials endowed by nano-twinned CuAgSe secondary phase. Acta Mater..

[16] Tee S.Y., Tan X.Y., Wang X., Lee C.J.J., Win K.Y., Ni X.P., Teo S.L., Seng D.H.L., Tanaka Y., Han M.-Y. (2022). Aqueous Synthesis, Doping, and Processing of n-Type Ag_2_Se for High Thermoelectric Performance at Near-Room-Temperature. Inorg. Chem..

[17] Jood P., Ohta M. (2020). Temperature-Dependent Structural Variation and Cu Substitution in Thermoelectric Silver Selenide. ACS Appl. Energy Mater..

[18] Zhou H., Zhang Z., Sun C., Deng H., Fu Q. (2020). Biomimetic Approach to Facilitate the High Filler Content in Free-Standing and Flexible Thermoelectric Polymer Composite Films Based on PVDF and Ag_2_Se Nanowires. ACS Appl. Mater. Inter..

[19] Ding Y., Qiu Y., Cai K., Yao Q., Chen S., Chen L., He J. (2019). High performance n-type Ag_2_Se film on nylon membrane for flexible thermoelectric power generator. Nat. Commun..

[20] Lei Y., Qi R., Chen M., Chen H., Xing C., Sui F., Gu L., He W., Zhang Y., Baba T. (2022). Microstructurally Tailored Thin β-Ag_2_Se Films toward Commercial Flexible Thermoelectrics. Adv. Mater..

[21] Gao J., Miao L., Lai H., Zhu S., Peng Y., Wang X., Koumoto K., Cai H. (2020). Thermoelectric Flexible Silver Selenide Films: Compositional and Length Optimization. iScience.

[22] Zheng Z.-H., Li Y.-L., Niu J.-Y., Wei M., Zhang D.-L., Zhong Y.-m., Nisar M., Abbas A., Chen S., Li F. (2022). Significantly (00l)-textured Ag_2_Se thin films with excellent thermoelectric performance for flexible power applications. J. Mater. Chem. A.

[23] Liu Y., Lu Y., Wang Z., Li J., Wei P., Zhao W., Chen L., Cai K. (2022). High performance Ag_2_Se films by a one-pot method for a flexible thermoelectric generator. J. Mater. Chem. A.

[24] Jiang C., Ding Y., Cai K., Tong L., Lu Y., Zhao W., Wei P. (2020). Ultrahigh Performance of n-Type Ag_2_Se Films for Flexible Thermoelectric Power Generators. ACS Appl. Mater. Int..

[25] Lu Y., Qiu Y., Cai K., Ding Y., Wang M., Jiang C., Yao Q., Huang C., Chen L., He J. (2020). Ultrahigh power factor and flexible silver selenide-based composite film for thermoelectric devices. Energy Environ. Sci..

[26] Lu Y., Qiu Y., Cai K., Li X., Gao M., Jiang C., He J. (2020). Ultrahigh performance PEDOT/Ag_2_Se/CuAgSe composite film for wearable thermoelectric power generators. Mater. Today Phys..

[27] Jiang C., Wei P., Ding Y., Cai K., Tong L., Gao Q., Lu Y., Zhao W., Chen S. (2021). Ultrahigh performance polyvinylpyrrolidone/Ag_2_Se composite thermoelectric film for flexible energy harvesting. Nano Energy.

[28] Li Y., Lou Q., Yang J., Cai K., Liu Y., Lu Y., Qiu Y., Lu Y., Wang Z., Wu M. (2021). Exceptionally High Power Factor Ag_2_Se/Se/Polypyrrole Composite Films for Flexible Thermoelectric Generators. Adv. Funct. Mater..

[29] Wang Z., Gao Q., Wang W., Lu Y., Cai K., Li Y., Wu M., He J. (2021). High performance Ag_2_Se/Ag/PEDOT composite films for wearable thermoelectric power generators. Mater. Today Phys..

[30] Gao Q., Wang W., Lu Y., Cai K., Li Y., Wang Z., Wu M., Huang C., He J. (2021). High Power Factor Ag/Ag_2_Se Composite Films for Flexible Thermoelectric Generators. ACS Appl. Mater. Inter..

[31] Lu Y., Liu Y., Li Y., Cai K. (2021). The influence of Ga doping on preparation and thermoelectric properties of flexible Ag_2_Se films. Compos. Commun..

[32] Li X., Lu Y., Cai K., Gao M., Li Y., Wang Z., Wu M., Wei P., Zhao W., Du Y. (2022). Exceptional power factor of flexible Ag/Ag_2_Se thermoelectric composite films. Chem. Eng. J..

[33] Chen D., Tang K., Shen G., Sheng J., Fang Z., Liu X., Zheng H., Qian Y. (2003). Microwave-assisted synthesis of metal sulfides in ethylene glycol. Mater. Chem. Phys..

[34] Ni D., Chen Y., Yang X., Liu C., Cai K. (2018). Microwave-assisted synthesis method for rapid synthesis of tin selenide electrode material for supercapacitors. J. Alloys Compd..

[35] Pei J., Chen G., Jia D., Jin R., Xu H., Chen D. (2013). Rapid synthesis of Ag_2_Se dendrites with enhanced electrical performance by microwave-assisted solution method. N. J. Chem..

[36] Kojda D., Mitdank R., Handwerg M., Mogilatenko A., Albrecht M., Wang Z., Ruhhammer J., Kroener M., Woias P., Fischer S.F. (2015). Temperature-dependent thermoelectric properties of individual silver nanowires. Phys. Rev. B.

[37] Kishimoto S., Hasegawa T., Kinto H., Matsumoto O., Iida S. (2000). Effect and comparison of co-doping of Ag, Ag+In, and Ag+Cl in ZnS: N/GaAs layers prepared by vapor-phase epitaxy. J. Cryst. Growth.

