# Supplementary material for: High-Performance Ag2Se Film by a Microwave-Assisted Synthesis Method for Flexible Thermoelectric Generators
Source: Molecules. 2023 Sep 1;28(17):6397. doi: 10.3390/molecules28176397 (PMC10489907; doi:10.3390/molecules28176397)
Supplement: Supplementary file 1 [file molecules-28-06397-s001.zip › molecules-2565415-supplementary.pdf]

Supplementary Materials

High-Performance Ag2Se Film by a Microwave-Assisted Synthesis Method for Flexible Thermoelectric Generators

Zixing Wang, Ying Liu, Jiajia Li, Changjun Huang and Kefeng Cai *

Key Laboratory of Advanced Civil Engineering Materials of Ministry of Education, Shanghai Key Laboratory of Development and Application for Metal-Functional Materials, School of Materials Science & Engineering, Tongji University, Shanghai 201804, China; wzxing2023@163.com (Z.W.); liuying_polymer@163.com (Y.L.); sduljj2018@163.com (J.L.); hcj@tongji.edu.cn (C.H.)

* Correspondence: kfcai@tongji.edu.cn

Note S1: Synthesis of Ag NWs

In a typical procedure, 2 ml 600 mM $FeCl_3 \cdot 6H_2O$ EG、2 ml 600 mM $CuCl_2 \cdot 2H_2O$ EG、0.800 g PVP K-30, and 0.0568 g SDBS were added into 75 ml EG in a 200 ml glass beaker with stirring slowly. After fully dissolving, 1.2178 g $AgNO_3$ was added to the solution with stirred slowly. Then, the beaker with the obtained solution was put in a domestic microwave oven. The solution was heated with a power of ~ 350 W for 4 min. After the reaction, centrifugation was used to collect the obtained yellow products and then washed with alcohol and distilled water alternately several times. Finally, the as-prepared Ag NWs were dispersed into 200 mL EG and a suspension was formed for further synthesis reactions.

Note S2

Assembly of the flexible TE Generator

The f-TEG consists of six strips (20 mm×5 mm× 22.5 μm) of the Ag_2Se film. The six strips were stuck on a PI substrate with an interval of 5 mm. The two ends of each strip were coated with a thin layer of Au to reduce the contact resistance. Then, each strip was connected by Ag paste (SPI#04998-AB) in series.

Note S3

TE property measurement and structure characterization

The crystallinity and phase composition of Ag NWs, Ag_2Se film before hot pressing and Ag_2Se film after hot pressing were examined by X-ray diffraction (XRD) (D/MAX2550VB3+/PCII). Field-emission scanning electron microscopy (FEI Nova

NanoSEM 450) was used to observe the surface morphology of Ag NWs, Ag_2Se powder and Ag_2Se film. X-ray photoelectron spectroscopy (XPS) was obtained by a Thermo Scientific K-Alpha. A transmission electron microscope (TEM, JEM-2100F) was used to investigate the internal details of the film. The Seebeck coefficient at RT was measured by the slope of the linear relationship between the thermal electromotive force and temperature difference between two ends on one side of each film. The temperature-dependent TE properties were measured by a Cryoall CTA-3 instrument in He atmosphere, with an instrument test error of ±5% for both σ and S. The Hall coefficient was measured by the Hall measurement system (LakeShore 8404).

The flexible test of the Ag_2Se film was performed by bending the film along a 4 mm radius rod for fixed times and then the resistance of each bending was measured. The flexibility of the film is assessed by observing the difference between the resistance value after bending (R) and the initial resistance (R_0).

For the output performance test of the f-TEG, all circuit components were connected with conducting wires according to the circuit diagram. In the TE generator, heat transfers from the hot side to the cold side along the length direction of the TE legs. We used an automatic temperature-controlling system to heat a copper block and one end of the f-TEG was put on the copper block as the hot side (T + ΔT). The other end of the f-TEG was put on an adiabatic foam acting as the cold side (T). The temperature of two ends was measured by two thermocouples. The f-TEG was connected as a generator into the circuit, which also includes a variable resistance box and an ammeter. Then we collected the output voltage and current by adjusting the variable resistance box at a particular temperature difference. The temperature difference was varied by setting different heating temperatures.

Note S4: Theoretical calculation details

We used the single parabolic band (SPB) model to calculate the Pisarenko curve and electronic thermal conductivity (κ_e). The solutions of the various transport properties in the SPB model involve integrals that are represented in terms of the "Fermi-Dirac" integral: "Fermi-Dirac" integral

$$F_i(\eta)\int_0^{\infty} f\varepsilon^i d\varepsilon = \int_0^{\infty} \frac{\varepsilon^i}{1+\exp\,(\varepsilon-\eta)} d\varepsilon$$

where $F_i(\eta)$ is the Fermi integral of order i, ε represents the reduced energy (E_F/k_BT) and η the reduced Fermi level (E_F/k_BT), i is the index of the integral, depends on both the transport property and energy dependence of the relaxation time of the charge carriers.

Within the SPB model, the transport parameters are expressed as follows:

Seebeck coefficient (S)

$$S = \frac{k_B}{e}\left(\frac{2F_1(\eta)}{F_0(\eta)} - \eta\right)$$

Electrical conductivity (σ)

$$\sigma = n_H \mu_H e$$

Hall carrier concentration (n_H)

$$n_H = \frac{4\pi}{r_H}\left(\frac{2m^* k_B T}{h^2}\right)^{\frac{2}{3}} F_{\frac{1}{2}}(\eta)$$

where $m*$ is the density of states effective mass, k_B is Boltzmann's constant, h is Planck's constant, r_H is the Hall factor for acoustic phonon scattering, which can be given by

$$r_H = \frac{3}{4}\frac{F_{\frac{1}{2}}(\eta)F_{-\frac{1}{2}}(\eta)}{F_0^2(\eta)}$$

Lorenz number (L)

$$L = (\frac{k_B}{e})^2 \frac{3F_0(\eta)F_2(\eta) - 4F_1^2(\eta)}{F_0^2(\eta)}$$

Electrical thermal conductivity (κ_e)

$k_e = L\sigma T$

Lattice thermal conductivity (κ_l)

$k_l = k - k_e$

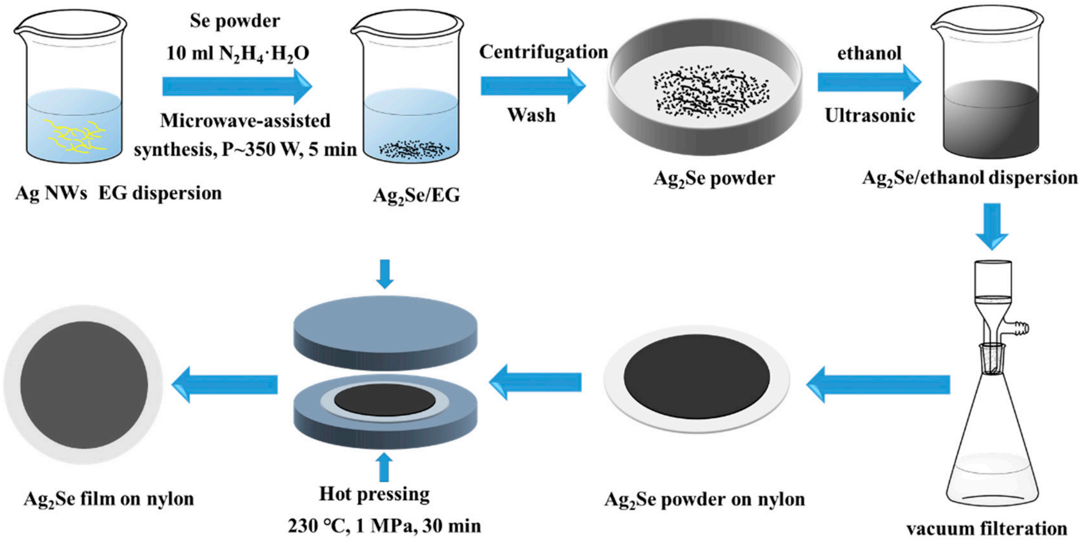

Figure S1. Schematic demonstrating the preparation process of the Ag_2Se film

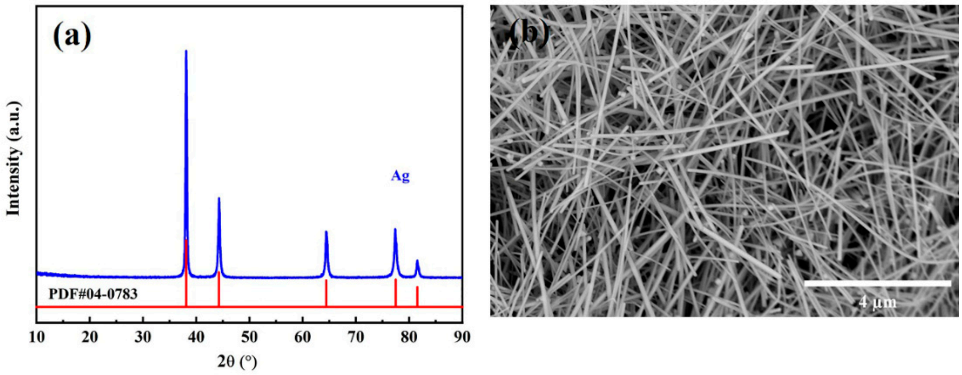

Figure S2. Characterization of the Ag NWs. (a) XRD pattern of Ag NWs, (b) surface FESEM image of Ag NWs

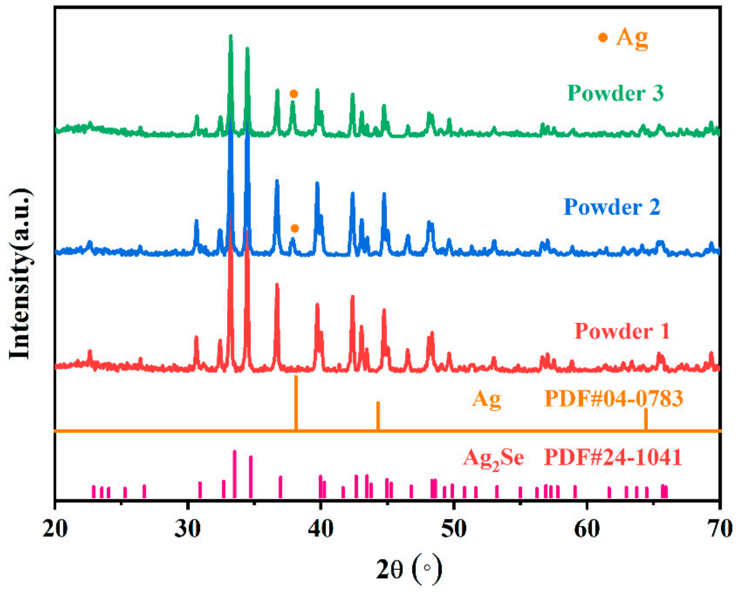

Figure S3. XRD patterns of powders 1–3.

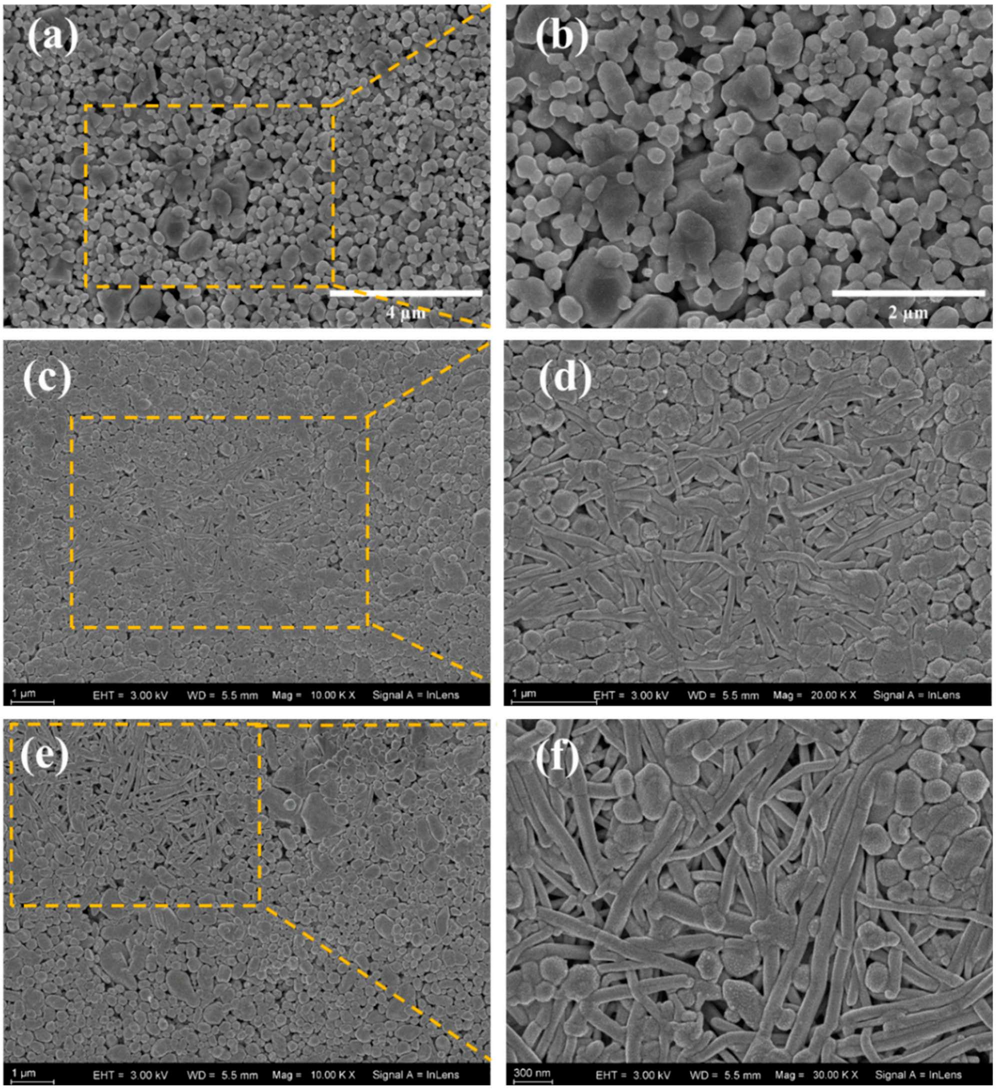

Figure S4. Surface FESEM images of powders 1–3: (a–b) powder 1, (c–d) powder 2, (e–f) powder 3.

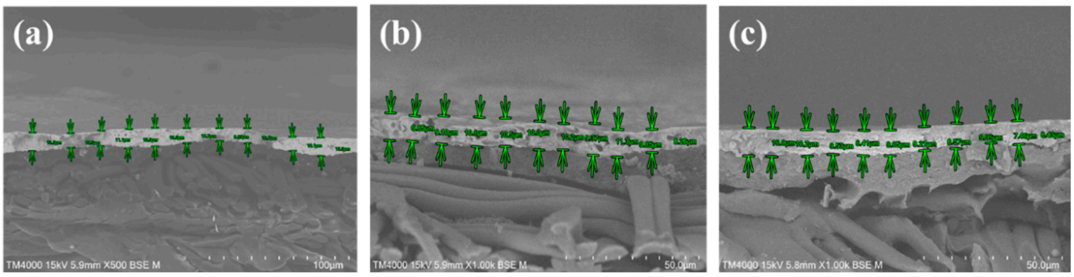

Figure S5. Cross-sectional SEM images of films 1–3: (a) film 1, (b) film 2, (c) film 3.

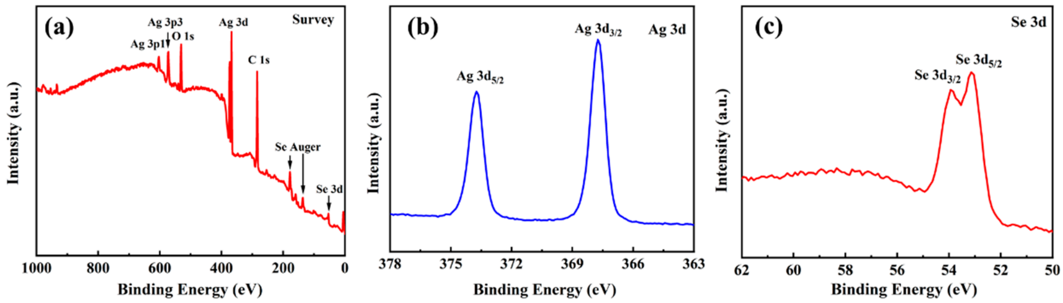

Figure S6. XPS analysis of film 1. (a) XPS survey spectrum. (b) Ag 3d spectrum. (c) Se 3d spectrum.

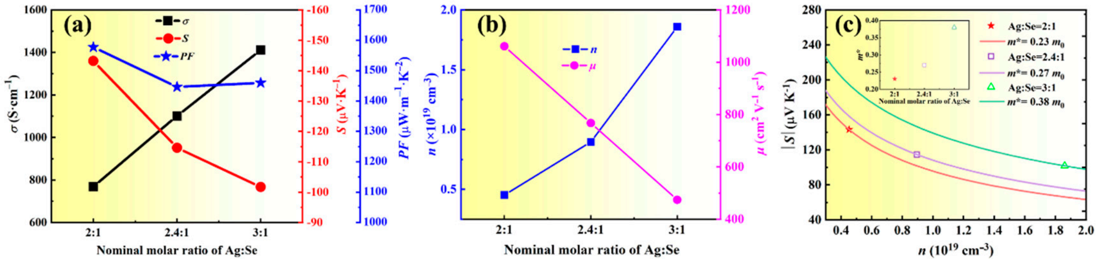

Figure S7. TE performance at RT of films 1–3 as functions of nominal the molar ratios of Ag/Se. (a) S, σ, and PF. (b) n and μ. (c) Hall carrier concentration dependence of the Seebeck coefficient with estimated DOS effective masses for the three films.

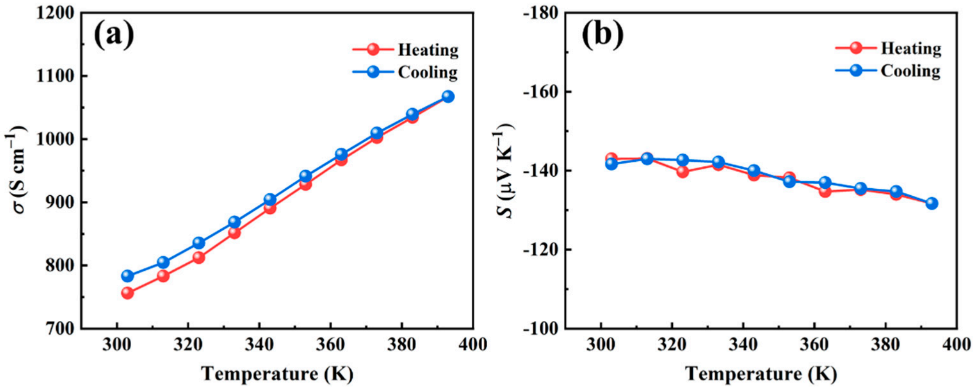

Figure S8. Temperature dependence of the TE performance of film 1 for the heating and cooling cycle. (a) σ, (b) S.

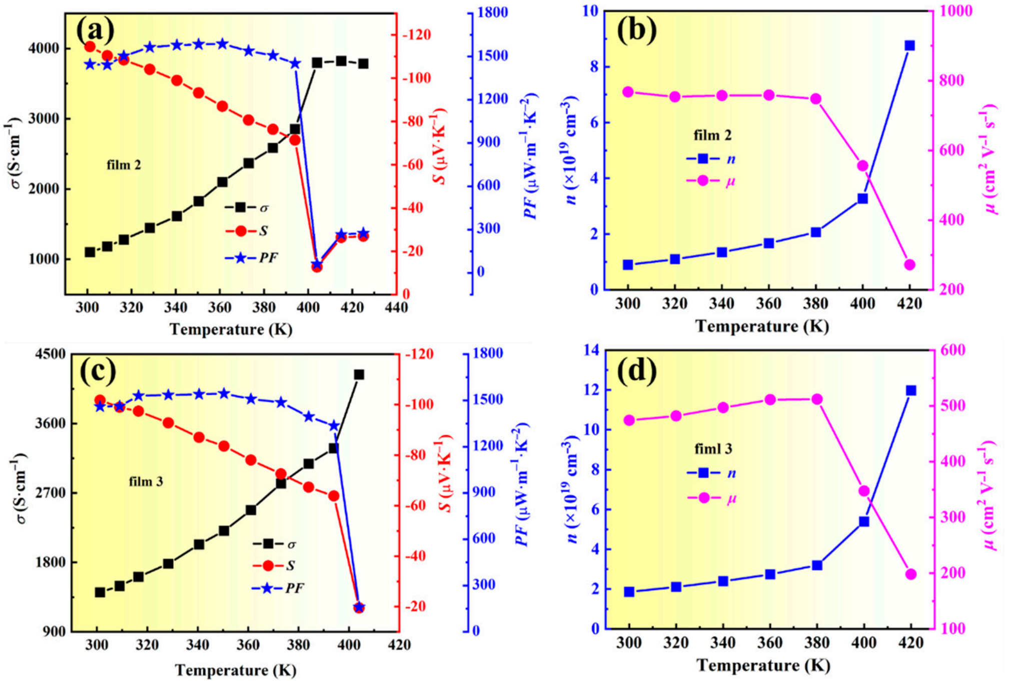

Figure S9. TE performance with varying temperature for films 2 and 3. (a) S, σ, and PF of film 2. (b) n and μ of film 2. (c) S, σ, and PF of film 3. (d) n and μ of film 3.

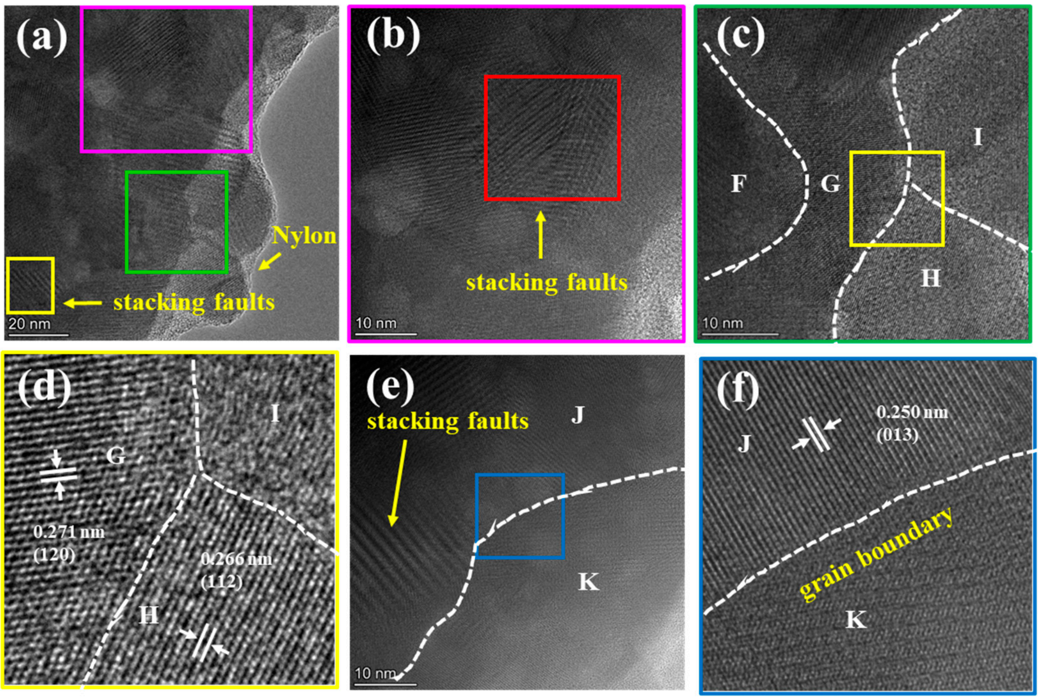

Figure S10. Internal microstructure of film 1. (a) Representative TEM image including a few nanograins. (b) HRTEM image corresponding to the pink square marked in (a). (c) HRTEM images corresponding to the green square marked in (a). (d) Enlarged image of the yellow square marked in (c). (e) HRTEM image of another two adjacent grains. (f) Enlarged image of the blue square marked in (e).

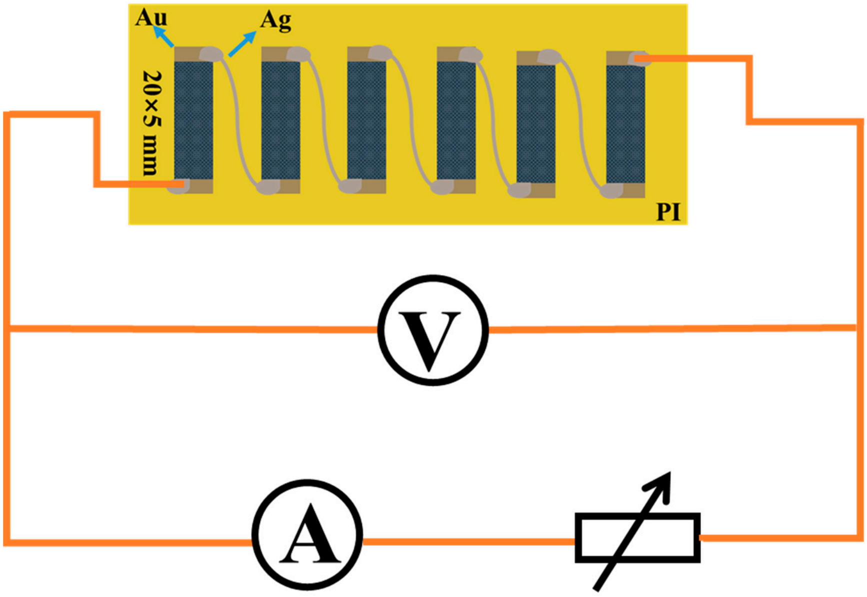

Figure S11. A schematic diagram for the output performance measurement of the f-TEG

Table S1. Comparison of the two methods of synthesis for Ag_2Se powder

Method	Experimental procedure	Time	Solvent	Total time	Ref.
	Synthesis of Se NWs	4 h	DI water		
	Washing of Se NWs	3 h	Ethanol, DI water		
Se NW	Growth of Se NWs	12 h	Ethanol	45 h	[1–4]
Template	Drying of Se NWs	12 h			
	Dispersion of Se NWs	12 h	EG		
	Synthesis of Ag_2Se	2 h	EG		
	Synthesis of Ag NWs	4 min	EG		
Ag NW	Washing of Ag NWs	3 h	Ethanol, DI water		This
Template	Dispersion of Ag NWs	12 h	EG	15 h	work
	Synthesis of Ag_2Se	5 min	EG		

References

1. Ding, Y.; Qiu, Y.; Cai, K.; Yao, Q.; Chen, S.; Chen, L.; He, J. High performance n-type Ag_2Se film on nylon membrane for flexible thermoelectric power generator. *Nat. Commun.* **2019**, *10*, 841.
2. Jiang, C.; Ding, Y.; Cai, K.; Tong, L.; Lu, Y.; Zhao, W.; Wei, P. Ultrahigh Performance of n-Type Ag_2Se Films for Flexible Thermoelectric Power Generators. *ACS Appl. Mater. Inter.* **2020**, *12*, 9646–9655.
3. Jiang, C.; Wei, P.; Ding, Y.; Cai, K.; Tong, L.; Gao, Q.; Lu, Y.; Zhao, W.; Chen, S. Ultrahigh performance polyvinylpyrrolidone/Ag_2Se composite thermoelectric film for flexible energy harvesting. *Nano Energy* **2021**, *80*, 105488.
4. Lu, Y.; Qiu, Y.; Cai, K.; Li, X.; Gao, M.; Jiang, C.; He, J. Ultrahigh performance PEDOT/Ag_2Se/CuAgSe composite film for wearable thermoelectric power generators. *Mater. Today Phys.* **2020**, *14*, 100223.
